# How to Change Young Children’s Physical Activity and Sedentary Behavior: Mechanisms of Behavior Change in the INFANT Cluster Randomized Controlled Trial

**DOI:** 10.3390/children8060470

**Published:** 2021-06-02

**Authors:** Kylie D. Hesketh, Konsita Kuswara, Gavin Abbott, Jo Salmon, Jill A. Hnatiuk, Karen J. Campbell

**Affiliations:** Deakin University, Institute for Physical Activity and Nutrition, Geelong 3220, Australia; k.kuswara@deakin.edu.au (K.K.); gavin.abbott@deakin.edu.au (G.A.); jo.salmon@deakin.edu.au (J.S.); jill.hnatiuk@deakin.edu.au (J.A.H.); karen.campbell@deakin.edu.au (K.J.C.)

**Keywords:** early childhood, mediators, movement behaviors, intervention, television viewing, active play

## Abstract

Background: Understanding the mechanisms (mediators) of behavior change is crucial to designing more effective interventions. However, this is rarely reported. This paper investigates the mechanisms that explain the lack of intervention effect on physical activity and the significant effect on television viewing time from an early childhood trial. Methods: Secondary analyses were undertaken of data from a cluster randomized controlled trial. The Melbourne Infant Feeding, Activity and Nutrition Trial (INFANT) was a 15-month group program promoting obesity-protective behaviors from the age of 4 months. Outcomes relevant to the current study were child physical activity (accelerometer), television viewing time (maternal report) and 12 potential mediator scales (maternal report). Linear regression models used the product of coefficients method with a joint significance test. Results: Complete data were from 398 mother-child dyads. Despite weak evidence of an intervention effect on the mother’s physical activity knowledge and optimism, there was no effect on children’s physical activity, and no clear mechanisms were identified. An intervention effect was observed for the mothers’ television knowledge (unstandardized regression coefficient for *a* path (*a*) = 0.34, 95% confidence interval (CI_95_) = 0.22, 0.45), with weak evidence for maternal efficacy (*a* = 0.11, CI_95_ = −0.02, 0.24) and the use of television (*a* = −0.10, CI_95_ = −0.22, 0.01). The intervention impact on television knowledge explained 75% of the difference between the intervention and control groups in children’s television viewing. Conclusions: In the very early childhood period, as mothers are commencing their parenting journey, improving their behavioral knowledge appears to be the biggest contributor to reducing child television viewing, constituting a relatively simple strategy that could be implemented across clinical and public health settings. In contrast, it remains unclear what mechanisms may increase physical activity levels in this age group.

## 1. Introduction

The World Health Organization has identified childhood obesity as an urgent issue requiring intervention [1], along with key drivers of obesity including insufficient physical activity and excessive screen time [1,2]. Obesity prevention trials generally aim to improve obesity-promoting behaviors by targeting postulated mediating factors, the potential mechanisms of behavior change. However, few trials directly investigate these observed mechanisms, despite their importance for understanding why an intervention worked or not [3,4], which is information vital for the design of more effective interventions.

Interventions often target multiple behaviors and assume similar mechanisms of behavior change, yet findings from mediation studies suggest this is often not the case. Reviews of mediation analyses in obesity prevention trials among school-aged children showed specific mediation pathways for different behaviors [4,5]. For example, children’s self-efficacy and intention for behavior change were consistently reported to mediate changes in physical activity [4,5], but intrinsic motivation mediated sedentary behavior change [4,5].

In the burgeoning field of very early childhood (0–2 y) obesity prevention, the small number of trials to date have shown some success in reducing adiposity [6,7] and improving obesity-preventive behaviors [6,7,8,9]. While some have reported intervention effects on the postulated mediating factors [10,11], often referred to less explicitly as secondary or tertiary outcomes, few have investigated the mechanisms of behavior change.

The Melbourne Infant, Feeding, Activity and Nutrition Trial (INFANT), a 15-month early childhood obesity prevention program, aimed to promote healthy diets, physical activity and reduced sedentary behaviors in children from infancy by developing maternal knowledge, skills and confidence [12]. The main outcomes, reported previously [8], showed no impact on child adiposity or physical activity, but did show improvement in some measures of diet and reductions in television viewing time. Mechanisms of change for the children’s diet quality were found to be maternal feeding knowledge and the use of food as rewards [13]. This paper aims to investigate potential mechanisms relating to the other two outcomes: the lack of an observed intervention effect on children’s physical activity levels and a significant intervention effect on television viewing time.

## 2. Materials and Methods

### 2.1. Study Design

This study involved secondary mediation analyses of data from the INFANT cluster randomized controlled trial. INFANT was a 15-month, 6-session, face-to-face group program delivered by trained dietitians in first-time parent groups in Melbourne, Australia. Session information was supplemented with written information and a take-home DVD. The program aimed to promote healthy diet, physical activity and reduced sedentary behaviors from infancy by developing maternal knowledge, skills and confidence (the study protocol detailing randomization and procedures has been published elsewhere) [12]. Ethical approval was granted by the Deakin University Human Research Ethics Committee (EC 175-2007). The trial registration was as follows: ISRCTN Register ISRCTN81847050, registered 7 November 2007.

### 2.2. Participants

Recruitment (detailed elsewhere, including a CONSORT flowchart) [8,12] involved two-stage random sampling. First, 14 of 28 local government areas (LGAs) within a 60 km radius of the research center in Burwood, Victoria, Australia were randomly selected. Secondly, 50% (rounded up to an even number to ensure 1:1 randomization) of the first-time parent groups within each LGA were randomly selected. These groups were operated by the state-wide free universal Maternal and Child Health Service offered to all new mothers. Group eligibility required the consent of at least 8 mothers (6 in low socioeconomic LGAs). Declining or ineligible LGAs or groups were replaced by the next ones on the randomized list. The parents provided written informed consent.

### 2.3. Measures

Data collection (2008–2010) occurred at baseline (child age ~4 months) and program conclusion (child age ~20 months). With the exception of the children’s physical activity levels, data were gathered through maternal completed questionnaires.

#### 2.3.1. Participant Demographics

At baseline, each mother reported the child’s sex and date of birth and the mother’s education level (low (i.e., secondary school), medium (i.e., post-secondary trade or certificate) or high (i.e., university)), country of birth (Australia or other) and main language spoken at home (English or other). At the program’s conclusion, the mothers were retrospectively asked the age when their child began walking.

#### 2.3.2. Outcome Measures

Physical activity: At the program’s conclusion, the children wore ActiGraph accelerometers (Model GT1M, Pensacola, FL, USA), capturing data in 15 s epochs for 8 days excluding sleeping and water activities. A cut point of ≥192 counts per minute [14] was applied to the data to determine the minutes of total physical activity per day (light, moderate or vigorous intensity). For inclusion, usable accelerometer data were required for a minimum of 7.4 h/day for a minimum of 4 days. This provided acceptable reliability (ICC > 0.70) when using the Spearman–Brown prophecy formula for the total physical activity in this sample. [15] Accelerometry data were not collected at baseline as the children were preambulatory (4 mo old).

Television viewing: At baseline and the program’s conclusion, the mothers proxy reported the amount of time their child spent watching television in a typical day. Test-retest reliability assessment in a separate sample of 66 mothers of 4-month-olds and 51 mothers of 20-month-olds showed acceptable reliability (ICC = 0.69, CI_95_ = 0.54, 0.80, and ICC = 0.84, CI_95_ = 0.72, 0.90, respectively).

#### 2.3.3. Potential Mediators

The hypothesized mediators fit within the COM-B (capability, opportunity, motivation and behavior) behavior change framework [16]: maternal attitudes, knowledge and skills (capability), the home environment’s access to play equipment and space (opportunity) and maternal beliefs and intention (motivation). One potential mediator, future expectations, which comprised two items, was common across both outcomes. An additional six potential mediators of physical activity were derived from 37 items, and five were derived from 32 items for television viewing (Table 1). All items were reported by the mothers at baseline and the program’s conclusion, with the exception of facilitating physical activity and physical activity in the home environment, which were assessed only at the program’s conclusion as they were not relevant at baseline when the children were preambulatory. The items were purposely designed for this study due to a lack of validated measures for this age group. There was good internal consistency for the potential mediators (Cronbach’s alpha = 0.58–0.84 at baseline and 0.68–0.91 at the intervention’s conclusion) (Table 1). Test-retest reliability, assessed with a separate sample of mothers, indicated acceptable agreement [17] for most items (85% weighted kappa > 0.4 at baseline and 75% weighted kappa > 0.4 at the program’s conclusion).

#### 2.3.4. Statistical Analyses

To examine whether maternal and home environment factors mediated the intervention effects on children’s physical activity and television viewing, a series of linear regression models were utilized, following the product of coefficients method with a joint significance test [18,19]. As data were available from two time points, mediation was tested using a half-longitudinal framework [20]. Specifically, the models examined whether the intervention influenced potential mediators at the program’s conclusion while adjusting for baseline levels of mediators where available (*a* path) and whether these potential mediators were associated with children’s physical activity levels or television viewing behavior at the program’s conclusion (*b* path) (i.e., X1 → M2 → Y2). Following the MacArthur approach [21], the *b* path models initially included an interaction of the exposure and the mediator; if the *p*-value for the interaction term was <0.05, the interaction was omitted. For any potential mediators showing evidence of both *a* and *b* path associations at the *p* < 0.05 level, indirect effects were examined for single mediator models by testing the product of the *a* and *b* coefficients with bootstrapped, percentile-based 95% confidence intervals calculated [22]. To be conservative, indirect effects were also assessed for the potential mediators, showing evidence of *a* or *b* path associations at the *p* < 0.05 level and weak evidence (*p* < 0.1) for the other path. For each outcome, a multiple mediator model was tested, including all individual mediators whose indirect effect 95% confidence interval did not cross zero.

The models were adjusted for the child’s age, gender and maternal education, and they included cluster-robust standard errors to account for clustering within the groups of mothers. Models in which physical activity was the outcome were adjusted for the accelerometer wear time. Reported coefficients are unstandardized regression coefficients. Stata/SE 16·1 (StataCorp, College Station, TX, USA) was used to conduct all statistical analyses.

## 3. Results

Participants were recruited between June and December 2008, and post-intervention data were collected between September 2009 and March 2010. In total, 542/630 (86%) mother-child dyads across 62 parent groups participated, and 492 (91%) were retained to completion of the 15-month program. Details of participant recruitment and retention have been published elsewhere [8]. For current analyses, one dyad was excluded as the respondent was not a mother. Due to missing data for key variables, 93 participants were excluded. The final sample was 398 dyads. For analyses where physical activity was the outcome, 268 dyads with valid accelerometer data were included.

### 3.1. Participant Characteristics

At baseline, the mothers were aged M = 32.4 y (SD 4.2), born in Australia (81.2%) and primarily English-speaking (96.7%). Half (55.8%) of the mothers had tertiary education, with the remainder having completed secondary school (20.1%) or post-secondary trade or certificate qualifications (24.1%). Boys represented 53.8% of the children, and walking commenced at M = 13.2 mo (SD 2.0).

### 3.2. Physical Activity Outcome

There was no evidence of an intervention effect on the children’s physical activity time (path *c*) (*c* = −2.27, CI_95_ = −11.00, 6.47, *p* = 0.61). As shown in Table 2, there was weak evidence of an intervention effect on physical activity knowledge (*a* = 0.07, CI_95_ = 0.00, 0.14) and optimism (*a* = −0.10, CI_95_ = −0.18, −0.01). Mothers in the intervention group had greater knowledge of the importance of physical activity and the need for parents to facilitate it, but they also had lower optimism regarding their children’s ability and opportunities to engage in physical activity compared with the control group mothers. There was no evidence of an effect on the remaining potential mediators (path *a*).

When examining the effects of potential mediators on children’s physical activity time (path *b*), there was evidence of moderating effects of the intervention group for facilitating physical activity (*p* = 0.039), the physical activity home environment (*p* = 0.045) and future expectations (*p* = 0.024). Thus, these effects were estimated separately. For the control group, a positive association was observed for facilitating physical activity (*b* = 0.66, CI_95_ = 0.31, 1.00) and for the physical activity home environment (*b* = 2.86, CI_95_ = 0.20, 5.52) with physical activity time, and weak evidence for future expectations (*b* = 8.87, CI_95_ = −0.63, 18.36). For the intervention group, there was no evidence of associations between these potential mediators and physical activity. However, there was evidence of positive effects from physical activity knowledge (*b* = 18.61, CI_95_ = 3.49, 33.72) and physical activity optimism (*b* = 11.89, CI_95_ = 1.10, 22.68) on the children’s physical activity time for the sample overall.

Given at least weak evidence of an effect from the intervention on physical activity optimism and knowledge and an effect of these potential mediators on the physical activity outcome, the indirect effects (*a* ∗ *b*) for these two potential mediators were examined. The estimated indirect effects were −1.15 (CI_95_ = −3.01, 0.03) and 1.30 (CI_95_ = −0.10, 3.38), with the 95% confidence intervals crossing zero.

### 3.3. Television Viewing Outcome

An intervention effect on television viewing time (path *c*) was observed, with the intervention group children watching 14 min less television per day than the control group children (*c* = −14.35, CI_95_ = −27.95, −0.76). As is shown in Table 3, there was strong evidence of an intervention effect on television knowledge (*a* = 0.34, CI_95_ = 0.22, 0.45) and weak evidence for self-efficacy (*a* = 0.11, CI_95_ = −0.02, 0.24) and the use of television (*a* = −0.10, CI_95_ = −0.22, 0.01) (path *a*). The intervention group mothers indicated greater television knowledge (regarding recommendations and potential detriments of child viewing), greater self-efficacy for limiting their children’s television viewing and lower intended and actual use of television to entertain and distract their children compared with the mothers in the control group.

Higher maternal self-efficacy for limiting children’s television viewing (*b* = −30.33, CI_95_ = −40.56, −20.10), television knowledge (*b* = −32.28, CI_95_ = −41.34, −23.22) and expectations that their children, when older, would have similar physical activity and television habits to their own (*b* = −12.50, CI_95_ = −24.76, −0.25) were all associated with lower viewing times for the children. Higher television viewing was seen in children whose mothers had greater intended and actual use of television to entertain and distract (*b* = 35.75, CI_95_ = 21.86, 49.63) and provided greater facilitation of television viewing (*b* = 1.78, CI_95_ = 1.26, 2.30). The only potential mediator to not show evidence of an association with children’s television viewing times was sedentary behavior in the home environment (path *b*) (see Table 2).

Only television viewing knowledge showed evidence of effects at the *p* < 0.05 level for both the *a* and *b* path. Mediation analyses estimated an indirect effect of the intervention on children’s television viewing time via television viewing knowledge of -10.83 (CI_95_ = −15.78, −6.72) accounting for an estimated 75% of the total effect of the intervention on television viewing time. Both self-efficacy around television viewing (*p* = 0.086) and parent’s use of television (*p* = 0.071) showed weak evidence of an intervention effect on the potential mediator and strong evidence of an effect of the potential mediator on the television outcome (both *p* < 0.005). The indirect effect for self-efficacy was -3.43 (CI_95_ = −7.21, 0.54) and for use of television it was −3.70 (CI_95_ = −8.77, 0.16). In an exploratory multiple mediation model including these three potential mediators (results not shown), the estimated proportion of intervention effect on television viewing time accounted for was 76%.

## 4. Discussion

This study aimed to understand the mechanisms of behavior change (the *how*) associated with early childhood obesity prevention intervention for two behavioral outcomes: children’s physical activity, which showed no intervention effect, and television viewing, which was reduced by the intervention. This addresses a major evidence gap, as few studies progress beyond the investigation of *what* changed to understand *how* the behavior change occurred (or why it did not occur). Identifying the underlying mechanisms of behavior change is vital for both understanding how behaviors develop and how to modify them to improve health. Potentially explaining the lack of an intervention effect on children’s physical activity, there was a limited intervention impact on the physical activity mediating factors that were targeted. In contrast, the intervention appeared to have reduced children’s television viewing primarily by improving mothers’ knowledge about screen time recommendations and potential detriments to their children of television viewing from a young age, which explained three quarters of the total intervention effect.

Finding that maternal knowledge was the key mediator of the intervention’s impact on children’s television viewing time was somewhat unexpected. Typically, knowledge is considered necessary but insufficient for behavior change [23,24]. However, we similarly found that maternal feeding knowledge mediated the intervention effect on children’s diet quality in this sample [13]. It is likely these first-time mothers were starting at a low knowledge base regarding early childhood behaviors, and much of the information provided in the intervention was new to them [25]. Specifically for television viewing, while the American Academy of Pediatrics first issued a policy in 1999 encouraging parents to avoid television viewing for children under 2 years old [26], Australia only released guidelines for early childhood in 2009 [27] when INFANT was underway. Dissemination of the Australian guidelines was primarily through secondary sources such as health professionals. It is unclear how widely dissemination to parents was achieved. What is known is that less than one third of maternal and child health nurses, the key primary health care contact for mothers in Victoria, Australia through the first year after birth, reported routinely discussing screen time recommendations in their consults [28]. Many nurses suggest they do not feel comfortable raising this issue with families [28], and only 27% report having access to education materials on limiting screen time. It is possible that INFANT bridged this gap by providing mothers with such educational materials and knowledge alongside the strategies to enable it.

This study identified weak evidence that maternal efficacy for limiting a child’s television viewing and the use of television to distract and occupy their child may also be mediators. While these constructs showed strong associations with children’s television viewing times, as would be expected and the reason they were targeted, there was only weak evidence that the intervention had impacted them. This suggests that the impact of INFANT on children’s television viewing time, while significant both statistically and at a public health level [8], may have been even greater had the program succeeded in having a greater impact on these mediators. Further work is required to identify strategies to successfully increase maternal efficacy and decrease the use of television as a means to manage behavior or entertain a child while the mother undertakes other tasks, an occurrence mothers regularly report [29]. Given the now widespread use of a range of screen devices for young children, it is likely mothers will require even greater support to limit their children’s exposure, particularly as newer screen devices are more portable and readily accessible even outside the home.

Of note, all potential mediators of television viewing targeted in this intervention, with the exception of the home environment, showed strong evidence of an association with children’s television viewing time. This indicates that they were appropriate targets. In contrast, few potential mediators showed evidence of an association with children’s physical activity or of having been impacted by the intervention. This helps explain the lack of the intervention’s effect on children’s physical activity, suggesting both a lack of effect of the intervention on the targeted mediators and also the potential that the intervention targeted the wrong mediators. It is possible that the children in this study were too young for there to be clear associations with their physical activity levels yet; many had not been walking confidently for long. Other studies have reported that parents place less emphasis on influencing their children’s physical activity, assuming they will naturally be active [29]. This may have impacted the mothers’ engagement with the physical activity messages and strategies promoted in the intervention. Certainly, physical activity in very young children remains poorly understood, and this study further highlights the importance of comprehending the mechanisms at play.

The strengths of this study include the strong study design, which was based within a cluster randomized controlled trial with longitudinal data, enabling the assessment of causal relationships. However, as a half-longitudinal design was employed, it is not possible to draw unequivocal conclusions on causality, given that it would be assumed that the intervention’s effects on potential mediators would precede the effects on behaviors. A further strength is the comprehensive assessment of potential mediators and the objective assessment of children’s physical activity. A limitation is the constraint of trying to capture what are likely to be complex and dynamic processes occurring within families via simple survey-based measures. The proxy reporting of children’s television viewing time is a further limitation. However, in the absence of objective measures suitable for community-based studies, the measure employed was the best available with good test-retest reliability. While a focus on television viewing for this age group was appropriate at the time of the study’s development, there is now widespread use of portable screen devices among young children. Hence, future studies would be advised to focus on screen time more broadly. The response and retention rates were much higher than is typical for this type of research. Nonetheless, the sample was biased toward more educated mothers, which is common in community-based research.

In conclusion, this study suggests that, at least in the very early childhood period when the parenting journey is commencing, equipping mothers with the knowledge that television viewing does not confer health and developmental benefits and is not recommended for children under the age of two years may be the biggest contributor to reducing child viewing time. This constitutes a relatively easy intervention that could be implemented across a range of clinical and public health settings. Strengthening the support and skill-building aspects of this intervention to improve mothers’ confidence to limit their children’s television viewing and enact alternative strategies to keep their children entertained may further contribute to a significant reduction in television viewing time. Such strategies should be central to intervention efforts in this age group. In contrast, it is currently unclear what mechanisms are likely to increase the physical activity levels of children of this age. Given the strong public health imperative to instill physical activity from a young age, this remains a key area for further investigation.

## Figures and Tables

**Table 1:** Descriptions of potential mediator measures.

Measures	Description	Scale and Range	Baseline			Program Conclusion		
			Chronbach’s α	Control	Intervention	Chronbach’s α	Control	Intervention
				M ± SD	M ± SD		M ± SD	M ± SD
**Physical activity-related mediators**								
Self-efficacy around physical activity (3 items)	Parental confidence to promote active play (i.e., provide my child with a range of active play options).	4 points: 0 = not at all confident, 3 = very confident.	0.84	2.5 ± 0.5	2.5 ± 0.5	0.83	2.5 ± 0.5	2.5 ± 0.5
Facilitating physical activity ^a^ (6 items)	Over the past month, frequency of parents facilitating their child’s active play (e.g., by being active with them), providing active opportunities.	6 points: 0 = never or rarely, 1 = some days each week, 2 = most days each week, 3 = every day, 4 = at least once a day, 5 = several times each day. Recoded into times/week scores. Range = 0–84.	NA			0.73	41.8 ± 13.3	40·6 ± 13·7
Physical activity knowledge (10 items)	Agreement with statements on benefits and recommendations relating to children’s physical activity (e.g., parents need to encourage their babies and toddlers to be physically active).	4 points: 0 = strongly disagree, 3 = strongly agree.	0.80	2.5 ± 0.3	2.5 ± 0.3	0.83	2.5 ± 0.3	2.5 ± 0.4
Views on physical activity (4 items)	Agreement with statements on personal views on children’s physical activity (e.g., a placid and inactive child is easier to look after than an active one).	4 points: 0 = strongly disagree, 3 = strongly agree.	0.61	0.9 ± 0.5	0.9 ± 0.5	0.72	0.7 ± 0.5	0.6 ± 0.5
Physical activity home environment (11 items) ^a,c^	Indication of whether child has access to items or environment that promote physical activity (e.g., tricycle or safe and secure play space outside).	2 points: 0 = no, 1 = yes. Range = 0–11.	NA			NA	6.3 ± 2.0	6.2 ± 1.8
Physical activity optimism (3 items)	Agreement with statements relating to own child’s physical activity (e.g., it is easy for my child to get plenty of active play time every day).	4 points: 0 = strongly disagree, 3 = strongly agree.	0.72	2.3 ± 0.5	2.3 ± 0.4	0.78	2.4 ± 0.5	2.3 ± 0.4
Future expectations (2 items) ^b^	Agreement with statements relating to future activity levels and television habits (e.g., I think that when he or she is older, my child will have similar physical activity levels to my own).	4 points: 0 = strongly disagree, 3 = strongly agree.	0.58	1.7 ± 0.5	1.7 ± 0.5	0.68	1.9 ± 0.6	1.7 ± 0.6
**Television viewing-related mediators**								
Self-efficacy around television viewing (3 items)	Parental confidence to limit child’s television viewing (e.g., keep my child entertained without using TV, video or DVDs).	4 points: 0 = not at all confident, 3 = very confident.	0.73	2.0 ± 0.6	2.0 ± 0.6	0.83	2.2 ± 0.7	2.3 ± 0.6
Facilitating television viewing (7 items)	Over the past month, frequency of parents facilitating their child’s television viewing (e.g., turning the television on).	6 points: 0 = never or rarely, 1 = some days each week, 2 = most days each week, 3 = every day, 4 = at least once a day, 5 = several times each day. Recoded into times/week scores. Range = 0–98.	0.78	26.3 ± 15.6	27.0 ± 15.0	0.82	21.6 ± 15.2	21.4 ± 15.6
Television viewing knowledge (4 items)	Agreement with statements on detriments and recommendations relating to children’s television viewing activity (e.g., TV is educational for babies and toddlers).	4 points: 0 = strongly disagree, 3 = strongly agree (reverse scored).	0.87	1.3 ± 0.5	1.4 ± 0.5	0.91	1.3 ± 0.5	1.7 ± 0.7
Use of television (5 items)	Agreement with statements on how parents use television in relation to their child (e.g., I use TV to keep my child occupied so that I can get things done).	4 points: 0 = strongly disagree, 3 = strongly agree.	0.77	0.8 ± 0.5	0.8 ± 0.4	0.82	0.7 ± 0.5	0.6 ± 0.5
Sedentary behavior in home environment (13 items) ^c^	Indication of whether child has access to items or environment that promote screen use (e.g., DVD player).	0 = no, 1 = yes. Range = 0–13.	NA	4.9 ± 1.5	5.0 ± 1.6	NA	4.7 ± 1.6	4.8 ± 1.8
Future expectations (2 items) ^b^	Agreement with statements relating to future activity levels and television habits (e.g., I think that when he or she is older, my child will watch similar amounts of TV to me).	4 points: 0 = strongly disagree, 3 = strongly agree.	0.58	1.7 ± 0.5	1.7 ± 0.5	0.68	1.9 ± 0.6	1.7 ± 0.6

^a^ Measured at the program’s conclusion only. ^b^ Future expectations was included as a potential mediator for both outcomes. ^c^ Chronbach’s alpha not calculated for home environment measures as these were checklists. NA: not applicable.

**Table 2:** Mediating pathways between the intervention group, potential mediators and children’s physical activity time ^a^.

	Effects of Intervention on Potential Mediators ^b^		Effects of Potential Mediators on PA Outcome ^c^	
	*a* (95% CI)	*p*-Value	*b* (95% CI)	*p*-Value
Self-efficacy around physical activity	0.00 (−0.10, 0.10)	0.96	6·80 (−2.24, 15.85)	0.14
Facilitating physical activity	−1.32 (−4.60, 1.97)	0.43	Control group: 0.66 (0.31, 1.00) Intervention group: −0.03 (−0.60, 0.54)	<0.0005 0.92
Physical activity knowledge	0.07 (0.00, 0.14)	0.06	18.61 (3.49, 33.72)	0.02
Views on physical activity	−0.05 (−0.16, 0.05)	0.32	−2.18 (−11.92, 7.56)	0.66
Physical activity home environment	−0.09 (−0.56, 0.38)	0.71	Control group: 2.86 (0.20, 5.52) Intervention group: −2.30 (−6.64, 2.04)	0.04 0.29
Physical activity optimism	−0.10 (−0.18, −0.01)	0.03	11.89 (1.10, 22.68)	0.03
Future expectations	−0.06 (−0.20, 0.08)	0.43	Control group: 8·87 (−0.63, 18.36) Intervention group: −7.30 (−19.17, 4.56)	0.07 0.22

^a^ All analyses were adjusted for the child’s age and sex and maternal education. ^b^ Control group is a reference category. These models were additionally adjusted for baseline levels of potential mediators where available. Reported coefficients are unstandardized regression coefficients for path *a*. ^c^ These models were additionally adjusted for the intervention group, baseline levels of potential mediators where available and accelerometer wear time. Where there was evidence of an interaction between the intervention group and a potential mediator at the *p* < 0.05 level, separate effect estimates were presented for the two intervention groups. Reported coefficients are unstandardized regression coefficients for path *b*.

**Table 3:** Mediating pathways between the intervention group, potential mediators and children’s television viewing time ^a^.

	Effects of Intervention on Potential Mediators ^b^		Effects of Potential Mediators on TV Outcome ^c^	
	*a* (95% CI)	*p*-Value	*b* (95% CI)	*p*-Value
Self-efficacy around television viewing	0.11 (−0.02, 0.24)	0.086	−30.33 (−40.56, −20.10)	<0.0005
Facilitating television viewing	−0.99 (−3.98, 2.00)	0.51	1.78 (1.26, 2.30)	<0.0005
Television viewing knowledge	0.34 (0.22, 0.45)	<0.0005	−32.28 (−41.34, −23.22)	<0.0005
Use of television	−0.10 (−0.22, 0.01)	0.07	35.75 (21.86, 49.63)	<0.0005
Sedentary behavior in home environment	0.01 (−0.26, 0.28)	0.96	0.87 (−4.61, 6.36)	0.75
Future expectancies	−0.09 (−0.20, 0.03)	0.14	−12.50 (−24.76, −0.25)	0.046

^a^ All analyses were adjusted for the child’s age and sex and maternal education. ^b^ Control group is a reference category. These models were additionally adjusted for baseline levels of potential mediators. Reported coefficients are unstandardized regression coefficients for path *a*. ^c^ These models were additionally adjusted for the intervention group and baseline levels of potential mediators and child TV viewing times. Reported coefficients are unstandardized regression coefficients for path *b*.

## Data Availability

Deidentified data will be made available upon reasonable request and pending approval for release by the granting ethics committee.

## References

[1] World Health Organization (2016). Report of the Commission on Ending Childhood Obesity.

[2] World Health Organization (2019). Global Action Plan on Physical Activity 2018–2030: More Active People for a Healthier World.

[3] Brown H., Hume C., Pearson N., Salmon J. (2013). A systematic review of intervention effects on potential mediators of children’s physical activity. BMC Public Health.

[4] Kelly S., Stephens J., Hoying J., McGovern C., Melnyk B.M., Militello L. (2017). A systematic review of mediators of physical activity, nutrition, and screen time in adolescents: Implications for future research and clinical practice. Nurs. Outlook.

[5] Van Stralen M.M., Yildirim M., te Velde S.J., Brug J., van Mechelen W., Chinapaw M.J. (2011). What works in school-based energy balance behaviour interventions and what does not? A systematic review of mediating mechanisms. Int. J. Obes..

[6] Wen L.M., Baur L.A., Simpson J.M., Rissel C., Wardle K., Flood V.M. (2012). Effectiveness of home based early intervention on children’s BMI at age 2: Randomised controlled trial. BMJ.

[7] Daniels L.A., Mallan K., Nicholson J.M., Battistutta D., Magarey A. (2013). Outcomes of an Early Feeding Practices Intervention to Prevent Childhood Obesity. Pediatrics.

[8] Campbell K.J., Lioret S., McNaughton S.A., Crawford D.A., Salmon J., Ball K., McCallum Z., Gerner B.E., Spence A.C., Cameron A.J. (2013). A parent-focused intervention to reduce infant obesity risk behaviors: A ran-domized trial. Pediatrics.

[9] Taylor B.J., Gray A., Galland B.C., Heath A.-L.M., Lawrence J., Sayers R.M., Cameron S., Hanna M., Dale K., Coppell K.J. (2017). Targeting Sleep, Food, and Activity in Infants for Obesity Prevention: An RCT. Pediatrics.

[10] Daniels L.A., Mallan K., Battistutta D., Nicholson J.M., Perry R., Magarey A. (2012). Evaluation of an intervention to promote protective infant feeding practices to prevent childhood obesity: Outcomes of the NOURISH RCT at 14 months of age and 6 months post the first of two intervention modules. Int. J. Obes..

[11] Lioret S., Campbell K.J., Crawford D., Spence A.C., Hesketh K., McNaughton S.A. (2012). A parent focused child obesity prevention in-tervention improves some mother obesity risk behaviors: The Melbourne inFANT Program. Int. J. Behav. Nutr. Phys. Act..

[12] Campbell K., Hesketh K., Crawford D., Salmon J., Ball K., McCallum Z. (2008). The Infant Feeding Activity and Nutrition Trial (INFANT) an early intervention to prevent childhood obesity: Cluster-randomised controlled trial. BMC Public Health.

[13] Spence A.C., Campbell K.J., Crawford D.A., McNaughton S.A., Hesketh K.D. (2014). Mediators of improved child diet quality following a health promotion intervention: The Melbourne InFANT Program. Int. J. Behav. Nutr. Phys. Act..

[14] Trost S.G., Fees B.S., Haar S.J., Murray A.D., Crowe L.K. (2012). Identification and Validity of Accelerometer Cut-Points for Toddlers. Obes..

[15] Hnatiuk J., Ridgers N.D., Salmon J., Campbell K., Mccallum Z., Hesketh K. (2012). Physical Activity Levels and Patterns of 19-Month-Old Children. Med. Sci. Sports Exerc..

[16] Michie S., Van Stralen M.M., West R. (2011). The behaviour change wheel: A new method for characterising and designing behaviour change interventions. Implement. Sci..

[17] Cohen J. (1960). A Coefficient of Agreement for Nominal Scales. Educ. Psychol. Meas..

[18] Yzerbyt V., Muller D., Batailler C., Judd C.M. (2018). New recommendations for testing indirect effects in mediational models: The need to report and test component paths. J. Pers. Soc. Psychol..

[19] MacKinnon D.P., Lockwood C.M., Hoffman J.M., West S.G., Sheets V. (2002). A comparison of methods to test mediation and other in-tervening variable effects. Psychol. Methods.

[20] Cole D.A., Maxwell S.E. (2003). Testing Mediational Models With Longitudinal Data: Questions and Tips in the Use of Structural Equation Modeling. J. Abnorm. Psychol..

[21] Kraemer H.C., Kiernan M., Essex M., Kupfer D.J. (2008). How and why criteria defining moderators and mediators differ between the Baron & Kenny and MacArthur approaches. Health Psychol..

[22] Hayes A.F., Scharkow M. (2013). The relative trustworthiness of inferential tests of the indirect effect in statistical mediation analysis: Does method really matter?. Psychol. Sci..

[23] Kelly M.P., Barker M. (2016). Why is changing health-related behaviour so difficult?. Public Health.

[24] Worsley A. (2002). Nutrition knowledge and food consumption: Can nutrition knowledge change food behaviour?. Asia Pac. J. Clin. Nutr..

[25] Spence A.C., Hesketh K.D., Crawford D.A., Campbell K.J. (2016). Mothers’ perceptions of the influences on their child feeding practices—A qualitative study. Appetite.

[26] American Academy of Pediatrics (2011). Media Use by Children Younger Than 2 Years. Pediatrics.

[27] Department of Health and Aging (DoHA) (2009). Get Up and Grow: Healthy Eating and Physical Activity for Early Childhood. Physical Activity Guidelines for 0–5 Year Olds.

[28] Laws R., Campbell K.J., Van Der Pligt P., Ball K., Lynch J., Russell G., Taylor R., Denney-Wilson E. (2015). Obesity prevention in early life: An opportunity to better support the role of Maternal and Child Health Nurses in Australia. BMC Nurs..

[29] Hesketh K.D., Hinkley T., Campbell K.J. (2012). Children’s physical activity and screen time: Qualitative comparison of views of parents of infants and preschool children. Int. J. Behav. Nutr. Phys. Act..

